# Graft-versus-host disease affecting oral cavity. A review

**DOI:** 10.4317/jced.51975

**Published:** 2015-02-01

**Authors:** Maria Margaix-Muñoz, José V. Bagán, Yolanda Jiménez, María-Gracia Sarrión, Rafael Poveda-Roda

**Affiliations:** 1 DDS, PhD. Associate Professor of Oral Medicine. Department of Stomatology, University of Valencia. Valencia, Spain; 2 MD, DDS, PhD. Charmain of Oral Medicine. Department of Stomatology, University of Valencia. Head of Stomatology and Maxillofacial Surgery Service, University General Hospital of Valencia. Valencia, Spain; 3 MD, DDS, PhD. Assistant Professor of Stomatology. Department of Stomatology, University of Valencia. Valencia, Spain; 4 MD, DDS, PhD. Staff physician. Stomatology and Maxillofacial Surgery Service. Valencia University General Hospital. Valencia, Spain

## Abstract

Graft versus host disease (GVHD) is one of the most frequent and serious complications of hematopoietic stem cell transplantation, and is regarded as the leading cause of late mortality unrelated to the underlying malignant disease. GVHD is an autoimmune and alloimmune disorder that usually affects multiple organs and tissues, and exhibits a variable clinical course. It can manifest in either acute or chronic form. The acute presentation of GVHD is potentially fatal and typically affects the skin, gastrointestinal tract and liver. The chronic form is characterized by the involvement of a number of organs, including the oral cavity. Indeed, the oral cavity may be the only affected location in chronic GVHD. The clinical manifestations of chronic oral GVHD comprise lichenoid lesions, hyperkeratotic plaques and limited oral aperture secondary to sclerosis. The oral condition is usually mild, though moderate to severe erosive and ulcerated lesions may also be seen. The diagnosis is established from the clinical characteristics, though confirmation through biopsy study is sometimes needed. Local corticosteroids are the treatment of choice, offering overall response rates of close to 50%. Extracorporeal photopheresis and systemic corticosteroids in turn constitute second line treatment. Oral chronic GVHD is not considered a determinant factor for patient survival, which is close to 52% five years after diagnosis of the condition.

** Key words:**Chronic graft-versus-host disease, oral chronic graft-versus-host disease, pathogenics, management, survival.

## Introduction

Before transplantation, the recipient is subjected to myelosuppressive and immunosuppressive conditioning treatments in order to ensure that the recipient (host) immune system is reconstructed after grafting from the donor (graft) cells, thereby avoiding rejection problems. Following transplantation, however, the immunocompetent T lymphocytes of the donor may recognize as foreign the antigens expressed by the recipient cells - thereby triggering an immune reaction accompanied by intense inflammatory responses that result in damage to different organs and tissues of the recipient. This condition is known as graft versus host disease (GVHD), and is a consequence of the incompatibility between the HLA system antigens of the donor and recipient (1).

Allogenic hematopoietic stem cell transplantation (HSCT) is widely used for the treatment of different benign and malignant hematological diseases. In this context, GVHD is one of the most frequent and serious complications of HSCT, and is regarded as the leading cause of late mortality unrelated to the underlying disease process (2-4). Indeed, GVHD is one of the main reasons why HSCT is not used as often as would be desirable (5,6). GVHD is an autoimmune and alloimmune disorder that affects multiple organs and tissues and exhibits a variable course - adversely conditioning the life expectancy of those patients who develop the disorder (7). In the last 10 years, the incidence of acute GVHD (aGVHD) has remained constant, while that of chronic GVHD (cGVHD) appears to have increased (8). This has been a consequence of the growing use of hematopoietic cells instead of bone marrow transplants; the use of not fully HLA-compatible donors with or without blood ties to the recipient; the infusion of lymphocytes particularly in reduced intensity allotransplants; the number of transplants performed per year; and transplantation performed in increasingly older patients (7).

The acute form of GVHD is observed in 50-70% of all allogenic transplant patients, while chronic GVHD is seen in 30-50% (9). At present, the distinction between these two forms of GVHD is based more on the clinical characteristics in each case than on timing criteria as was common a few years ago (2,10). The acute presentation of GVHD is potentially fatal and typically affects the skin, gastrointestinal tract and liver (11). In the chronic form of GVHD the oral cavity is one of the most commonly affected regions, and may even be the sole body location affected by the disease (12). Chronic GVHD usually develops during the first three years after transplantation, and is normally preceded by the acute form. The clinical characteristics of cGVHD are similar to those of other immune mediated diseases such as lichen planus, lupus erythematosus or systemic sclerosis (10). Drug treatment for the prevention of aGVHD has no preventive effect upon cGVHD (7). On the other hand, there is no specific treatment for GVHD – the drugs of choice being corticosteroids followed by immune modulators (13,14).

The aim of the present study is to offer a practical update on oral chronic GVHD, fundamented on what we consider to be five key concerns in relation to this disease: (a) What are the risk factors for cGVHD and what is the pathogenic role of the T cells? (b) How often is the oral cavity affected in cGVHD? (c) In what cases would a biopsy be indicated to confirm the diagnosis of oral cGVHD? (d) Is the treatment of oral cGVHD effective? Does it depend on any known factor? (e) How does oral cGVHD evolve?

A Medline-PubMed and Cochrane Collaboration literature search (latest consultation: May 2014) was conducted to clarify these issues. The search comprised publications in English and Spanish, corresponding to studies conducted in humans, without time restrictions, and with the exclusion of isolated clinical cases.

## What are the risk factors for cGVHD and what is the pathogenic role of the T cells?

The described risk factors for cGVHD are HLA incompatibility or the absence of blood ties between the donor and recipient; advanced age of the donor and recipient; a female donor and male recipient; childbirth (parity) in female donors (allosensitization); the transplantation of mobilized peripheral blood cells; the infusion of donor lymphocytes; and antecedents of aGVHD (14-16). Advanced age, a female donor and male recipient, and the transplantation of mobilized peripheral blood cells appear to be particularly associated to cGVHD. Neither whole body irradiation nor the intensity of the conditioning treatment before trans-plantation appears to influence the appearance or not of cGVHD (15).

The immunopathogenic mechanism of the disease is not entirely clear, though it is known that donor T cell reactivity against the recipient tissues, in the form of exacerbated direct or indirect inflammatory responses, is the main triggering factor of GVHD (4,17). Although there are common risk factors, the existence of certain risk factors that are exclusive of each type of GVHD suggests that the underlying pathogenesis also differs among them. In oral cGVHD, activation of the interferon-1 pathway appears to play a role (7). According to other authors, the destruction of the thymus gland by alloreactive T cells could be the main triggering element in cGVHD, since it seems that cGVHD usually does not develop after autologous hematopoietic cell transplantation, and thymopoiesis and T cell renovation phenomena moreover may be observed (6). It has been suggested that aGVHD is characterized by a Th1 lymphocyte-mediated cellular immune response, while cGVHD is characterized by a Th2 lymphocyte-mediated humoral immune response (7).

Both the T cells and B lymphocytes are implicated in the loss of general immune tolerance. The role of the B lymphocytes in the pathogenesis of GVHD has been explored in recent publications, due to the capacity of these cells to produce antibodies, and the good response obtained with anti-CD20 drugs in other autoimmune disease processes with characteristics similar to those of GVHD (17). Patients with autoantibodies and cGVHD have more symptoms (7,18), though no specific autoantibody panel for cGVHD has been established. It has also been suggested that cytokines such as B cell activating factor (BAFF, belonging to the tumor necrosis factor family), and antigen-presenting cells (APCs) such as the dendritic cells, could also play a role (18).

**Figure 1:**
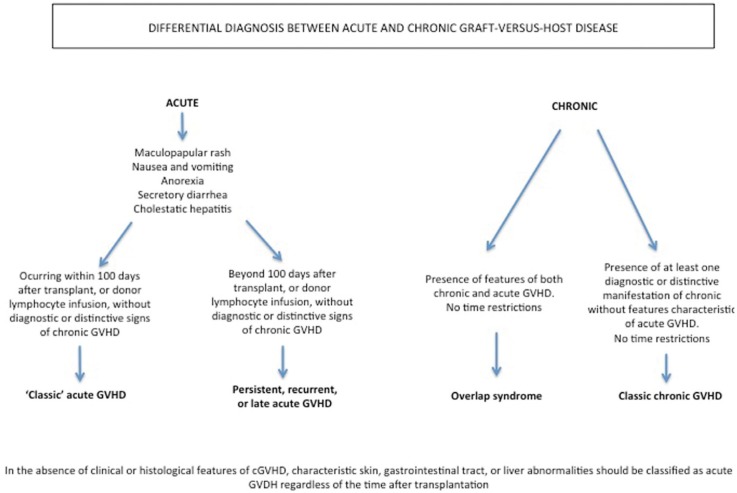
Diagnostic distinction between acute and chronic graft-versus-host disease (10).

The criteria that define and characterize the different forms of presentation of GVHD are shown in figure 1 and in  Table 1.

**Table 1:**
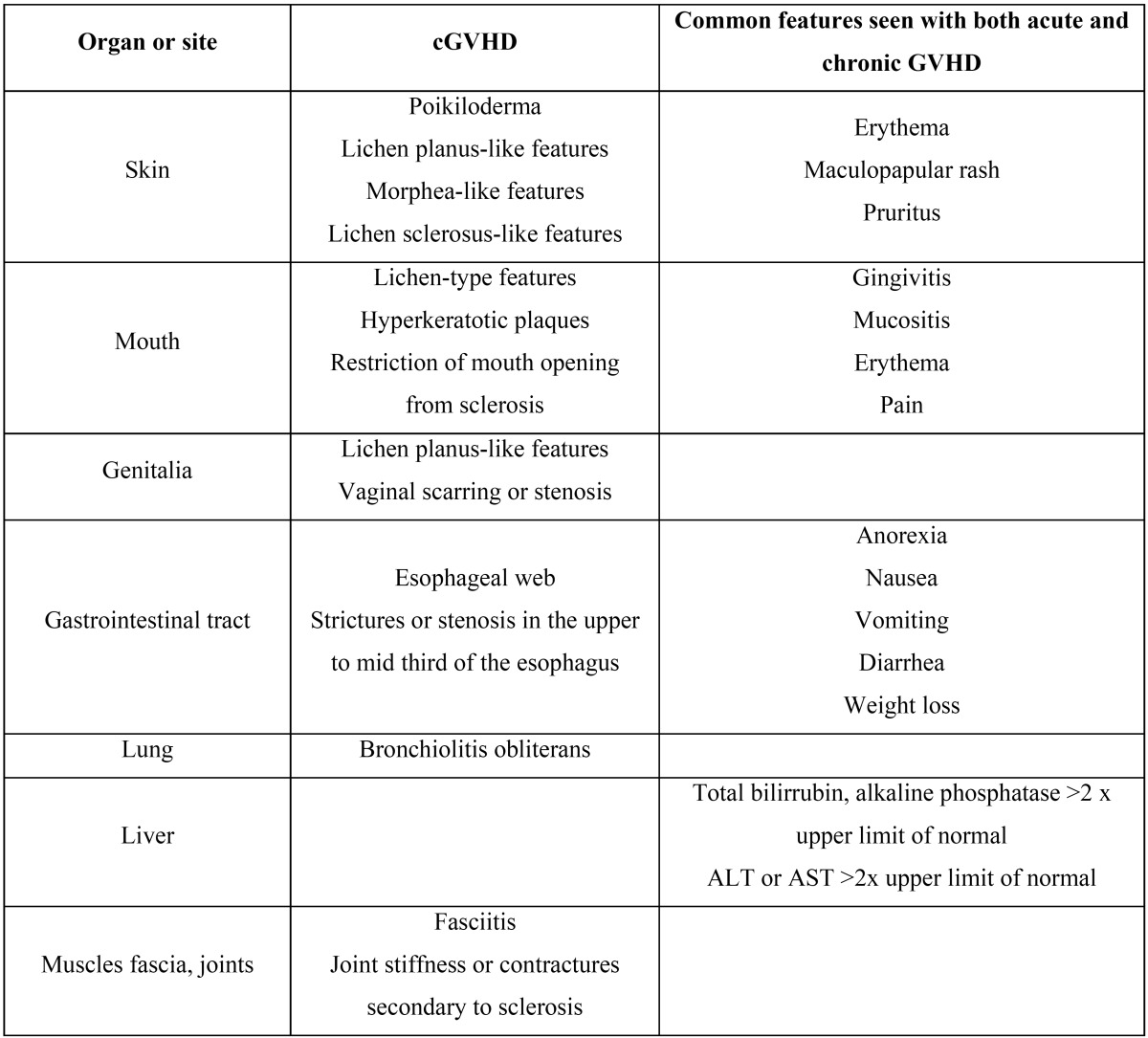
Diagnostic clinical manifestations of cGVHD (10).

## How often is the oral cavity affected in cGVHD?

Although the epidemiological information referred to GVHD is not homogeneous, it has been estimated that almost one-half of all patients subjected to hematopoietic stem cell transplantation will develop the disease (4,7). The prevalence of cGVHD among those who survive over 100 days after transplantation is 25-80% (2,14). In patients who have already developed cGVHD, the oral cavity is affected in 70% of those who have undergone hematopoietic stem cell transplantation and in 35% of those who have received a bone marrow transplant (14,19).

The prevalence of oral cGVHD ranges between 45-83% (18), and the oral cavity moreover may be the only affected body region (12).

The oral cavity, together with the skin, is one of the target organs of cGVHD, though manifestations are also commonly seen in the lungs, liver, genitals and gastrointestinal tract (2).

## In what cases would a biopsy be indicated to confirm the diagnosis of oral cGVHD?

**Figure 2:**
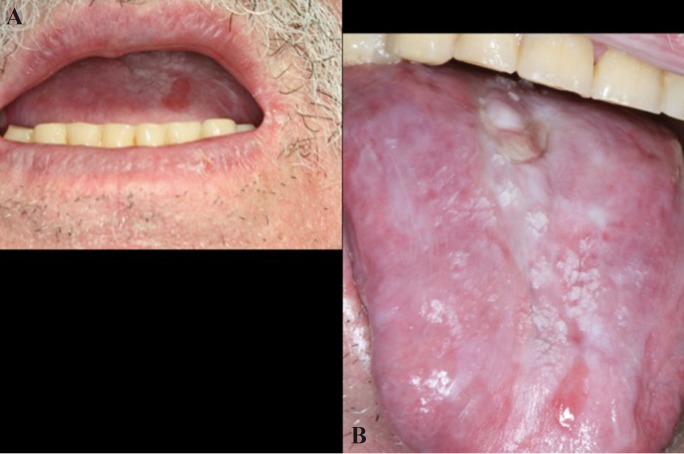
Clinical features of oral cGVHD. Mouth opening restriction due to sclerosis (A) and lichenoid lesions on tongue (B).

**Figure 3:**
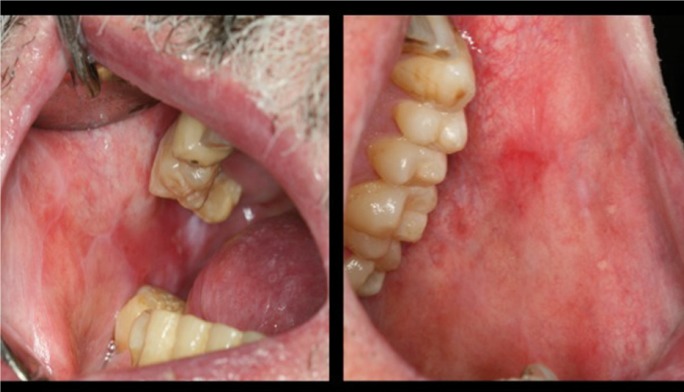
Clinical features of oral cGVHD. Bilateral lichenoid lesions affecting buccal mucosa.

The consensus document published in 2005 by the American Society for Blood and Marrow Transplantation (10) defines a series of general diagnostic criteria and specific differential features of oral cGVHD. The presence of these general diagnostic criteria would suffice to diagnose cGVHD (2,10), and comprise the appearance of clinical lichenoid lesions, hyperkeratotic plaques and limited oral aperture secondary to sclerosis (Figs. 2,3). The specific or distinctive clinical features in turn comprise xerostomia, the appearance of mucoceles, mucosal atrophy, pseudomembranes and ulcers; such manifestations alone are not enough to establish the diagnosis, however.

In the presence only of the abovementioned distinctive clinical features, the diagnosis of oral cGVHD could be established without the need for a biopsy provided we have radiological, histological or serological confirmation of the presence of cGVHD in other body organs. However, in the presence only of the distinctive clinical features without disease involvement in other organs, or if malignancy is suspected, an oral biopsy would be indicated with the purpose of establishing the diagnosis (17,20).

Histologically, cGVHD of the oral mucosa is characterized by the presence of dyskeratotic epithelial cells, apoptosis and an inflammatory infiltrate of lichenoid appearance beneath the epithelial basal lamina, consisting of CD3+ and CD68+ T cells (17,21). Fibrosis secondary to collagen deposits and atrophy are the differentiating features of cGVHD (14).

Other oral manifestations that can appear in both the acute and chronic presentation of the disease are mucositis, gingivitis, erythema and pain (10,22).

According to some authors and cGVHD study groups, the disease has similarities with other immune-mediated disorders such as lichen planus, lupus erythematosus, systemic sclerosis and Sjögren’s syndrome. In relation to this latter syndrome, and in the context of cGVHD, it has been reported that hyposalivation and xerostomia can manifest with some frequency as a consequence of progressive salivary gland atrophy. This in turn is associated to a worsening of patient quality of life referred to the oral cavity, a diminished body mass index, and more serious manifestations such as lung involvement (23).

The pediatric presentation of oral cGVHD is rarely characterized by dry mouth, dysgeusia or dysphagia (17). In contrast, it is common to observe erythema, lichenoid lesions, atrophy, mucoceles and pseudomembranous ulcers. Secondary infections produced by HSV-1, Coxsackie, HHV-6 and -7, and Enterovirus are also frequent. Phenomena such as alterations in dental root formation, microdontia, agenesis and malocclusion are considered long-term effects of HSCT in these patients (24).

With a view to standardizing the clinical, diagnostic and therapeutic criteria of oral cGVHD, the United States National Institutes of Health (NIH) in 2006 published a consensus document including a scale comprising a series of parameters that score the severity of cGVHD (mild, moderate, severe) and the response to treatment in the different affected areas of the oral cavity (25). The NIH also defined a scale allowing the patients to personally reflect their situation, quantifying pain, dry mouth and discomfort associated with the intake of liquid and solid foods. In some cases, the impression of the health professional does not coincide with that of the patient; in effect, patients typically describe a situation worse than that described by the health professional. The resolution of cGVHD is not always associated to a substantial change in patient quality of life, since the latter is also related to other factors such as the toxic effects of the treatment previously prescribed for the underlying malignant disease, the effects of immunosuppressors, and the problems inherent to the development of cGVHD (26). The presence of erythematous lesions and ulcers confers increased severity to oral cGVHD (9). Although the issue is subject to controversy, recent publications have vali-dated and reinforced the use of the NIH scale in the context of oral cGVHD (9,27).

## Is the treatment of oral cGVHD effective? Does it depend on any known factor?

Considering that no specific drug therapy for GVHD has yet been approved by the United States Food and Drug Administration (FDA)(4), the effectiveness of treatment can be expected to be limited. Furthermore, cGVHD and its treatment are associated to different complications such as secondary infections, osteoporosis, hypertension, hyperglycemia, renal failure and hyperlipidemia (28). In those patients who show a good response to initial treatment, the reactivation of cGVHD is observed in over one-half of the cases. This appears to be related to the severity of the disease (27).

Systemic therapy is indicated in severe cases where cGVHD affects a number of organs. Such treatment in turn can be complemented by local measures in patients with accessible lesions such as those of the skin and oral cavity.

The drugs of choice for the treatment of cGVHD are corticosteroids (class A recommendation, level of evidence Ia) with or without calcineurin inhibitors (C-1, IIa)(2-4,14,17,18). Prednisone is recommended at a dose of 1 mg/kg/day during two weeks, followed by 1 mg/kg on alternate days during four weeks if cGVHD remains stable or improves. In severe cases the recommendation is to administer 1 mg/kg/day during 2-3 months and then to lower the dose 10-20% each month for a total of 9 months (2).

When cGVHD is found to progress despite treatment with prednisone 1 mg/kg/day during two weeks; remains stable with ≥ 0.5 mg/kg/day of prednisone during 4-8 weeks; or when it is not possible to lower the dose to under 0.5 mg/kg of prednisone a day, the disease is considered to be refractory to corticosteroids and the second- and third-line treatment options are then used. Specifically, the second-line treatment options comprise extracorporeal photopheresis (extracorporeal exposure of peripheral blood mononuclear cells to UVA light-activated psoralen, followed by reinfusion of the treated cells to the patient) and drugs such as sirolimus, everolimus, pentostatin, rituximab and imatinib. Third-line treatment in turn comprises mofetil mycophenolate, methotrexate and corticosteroid pulses. Other described treatments for cGVHD have been less widely used and include hydroxychlo-roquine, clofazimine, cyclophosphamide, alemtuzumab, anti-TNFα drugs (infliximab, etanercept), thoraco-abdominal irradiation, thalidomide, alefacept, daclizumab/basiliximab, retinoids, azathioprine and mesenchymal stem cells (2).

Inamoto *et al.* (26), in a study of 283 patients with cGVHD, recorded an overall treatment response (complete remission + partial remission) of 32% during a follow-up period of 6 months. The organ-specific overall response rates were 45% in the case of the skin, 23% for the eyes, 32% for the oral cavity, 51% for the gastrointestinal tract, and 54% in the case of the liver. The treatments used were prednisone associated to a calcineurin inhibitor (46%), prednisone alone (22%), calcineurin inhibitors (18%), and other non-specified treatments (14%). Most of the patients presented cGVHD overlap syndrome (Fig. 1)(83%); more than one-half had previously suffered grade II-IV aGVHD; the incident and prevalent cases were similar in proportion (53% and 47%, respectively); and the most frequently affected organs were the oral cavity (61%) and skin. According to the mentioned authors, the scant overall response obtained may have been due to the fact that the treatment efficacy evaluation period was short. In this respect, it has been postulated that the results could improve over longer follow-up, since patient tolerance of systemic immunosuppressive therapy for cGVHD is generally reached after 2-3 years.

Treatment with rituximab (an anti-CD20 monoclonal antibody) is described as effective and safe, with very acceptable overall response rates, though its main inconvenience is the appearance of side effects. The pre- or peri-transplant administration of rituximab offers prevention against aGVHD but not against cGVHD. In this regard, it is believed that the early administration of rituximab could resolve cGVHD and moreover prevent the appearance of other manifestations inherent to the disease, though this is still only a hypothesis (29). Gutiérrez-Aguirre *et al.* (30) analyzed the effectiveness of low-dose alemtuzumab (an anti-CD52 monoclonal antibody) and rituximab in patients refractory to corticosteroid therapy. After one month of treatment, the overall response rate was 100% - a total of 67% of the patients achieving partial remission and 33% total remission of the disease. After 90 days, 50% presented partial remission, 28% complete remission, and 21% suffered reactivation of cGVHD. In this study the main affected body region was the oral cavity (86.7%). Kim *et al.* (31) administered weekly infusions of rituximab during four weeks, followed by a monthly infusion of rituximab during four months. Out of a total of 37 patients, 32 responded positively to the treatment, with a complete response in 8 cases and an incomplete response in 24. A total of 56.8% patients maintained the response to treatment during one year. The response was found to be greater (between 71.4-100%) in the case of the clinical manifestations of the skin, oral cavity and musculoskeletal system.

As first-line treatment, Wang *et al.* (32) used low-dose oral (10 mg) or parenteral methotrexate (15 mg) with or without immuno-suppressor therapy. After at least three doses of methotrexate, the overall response rate was 83%, with complete disease remission in 62% of the patients. In the multivariate analysis, the only variable associated to increased treatment response was found to be involvement of a single organ. The response to methotrexate appears to be particularly good in cases of skin involvement or disease affecting a single organ without concomitant thrombocytopenia.

As regards local treatment, none of the different drugs used for this purpose can be regarded as better than the rest, and their efficacy is moreover poor. Nevertheless, topical treatment in the form of corticosteroid rinses constitutes the first line of treatment for oral cGVHD (14).

As first-line therapy, Dignan *et al.* (20) propose a solution containing 0.5 mg of betamethasone in 10 ml of water retained in the oral cavity during two minutes, with repetition of administration three times a day. We can also use oral solutions of calcineurin inhibitors containing cyclosporine or tacrolimus. In resistant cases the suggested second-line treatment options are extracorporeal photopheresis and systemic corticosteroids.

In the review published by Meier *et al.* (17), on considering the local treatment options, a solution containing budesonide was proposed as first choice (class C1 recommendation, level of evidence III-3), with a reported overall response rate of 83%.

Park *et al.* (33) compared the effectiveness of budesonide (as a 0.03% aqueous solution) and dexamethasone (as a 0.01% aqueous solution), and recorded an overall response rate of 53.8% and 29.2%, respectively, after one month of follow-up. No statistically significant differences were recorded between the two drugs, though budesonide appeared to improve the pain reported by the patients.

The rest of topical treatment options for oral cGVHD are described in  Table 2 and  Table 3.

**Table 2:**
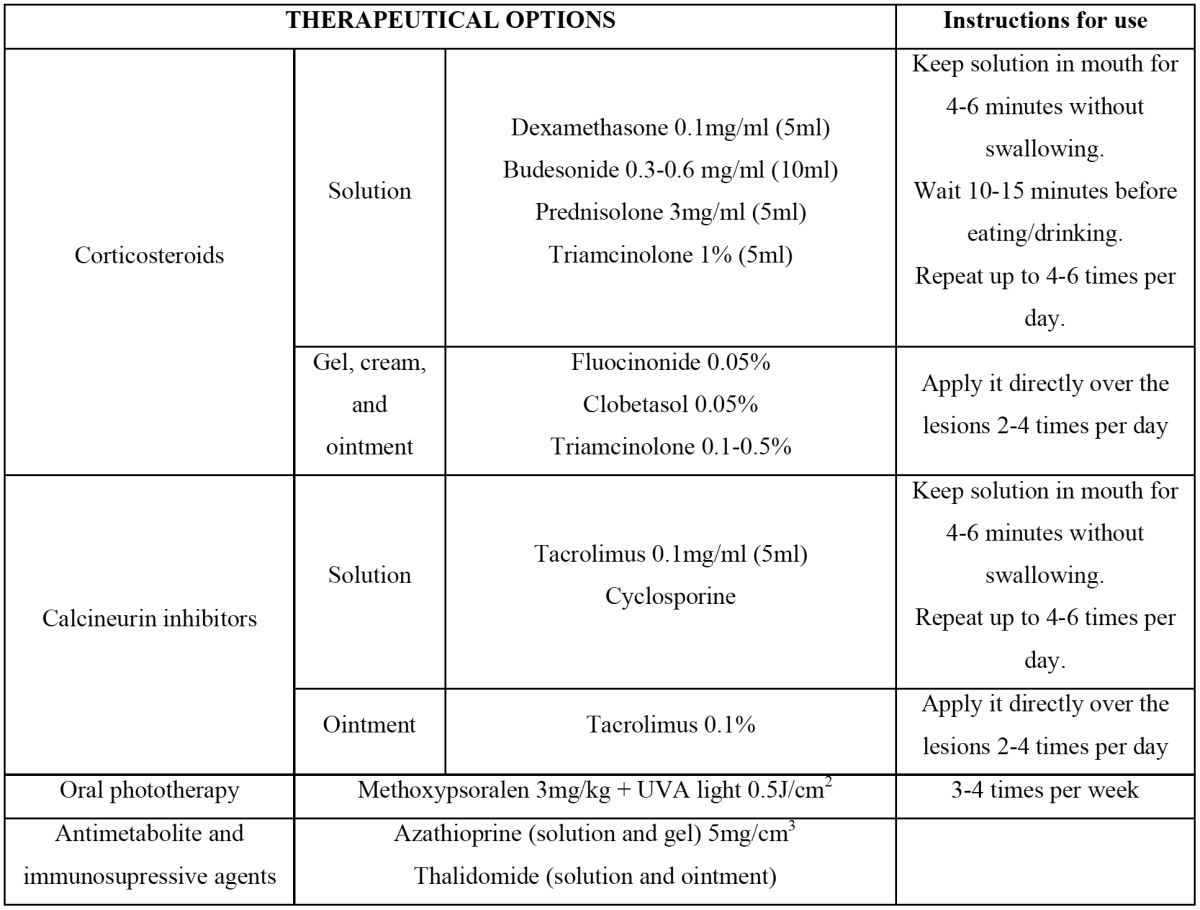
Topical management of oral mucosal cGVHD (1,17).

**Table 3:**
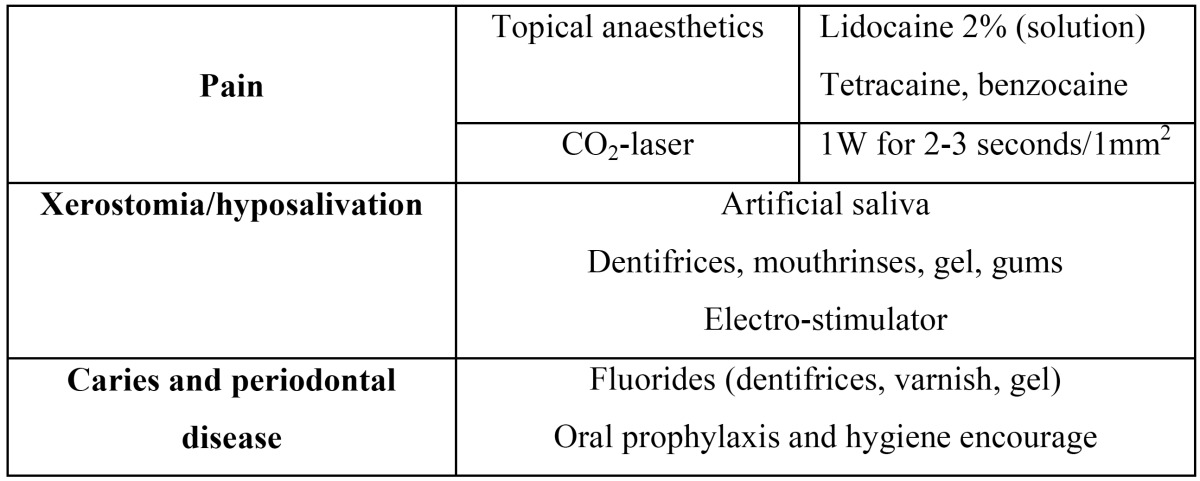
Topical supportive therapies for oral cGVHD (1,17,40).

## How does oral cGVHD evolve?

Chronic GVHD is the main cause of late patient mortality unrelated to malignant disease relapse (4,7,10), and is associated to important morbidity, a need for prolonged immunosuppressor therapy, functional disability, and impaired patient quality of life (1).

In most cases cGVHD proves extensive, affecting several organs, with moderate severity. The oral cavity is mostly characterized by milder disease (12,27).

The reported approximate overall survival rate is 76% three years (34) after the diagnosis of cGVHD, and 52% after 5 years (27). In pediatric patients the overall survival and disease-free survival rates 6 years after transplantation are 67% and 57%, respectively (35).

Two of the factors classically regarded as having the greatest impact upon survival are the progressive development of cGVHD from prior aGVHD, and the presence of thrombocytopenia (<100,000 platelets/μl). Other influencing factors are the involvement of multiple body organs or regions, hyperbilirubinemia, and extensive skin involvement at the time of the diagnosis (10).

Recent publications confirm the progressive development of cGVHD, the degree of severity, and liver and lung involvement as factors crucial to patient survival (27,34,36). Chronic overlap syndrome is likewise associated to lesser survival and greater functional disability (37).

Involvement of the joints, skin and lungs in cGVHD is what interferes most with patient functional capacity and worsens quality of life. As has been mentioned, cGVHD frequently manifests in the oral cavity, though its appearance in this location does not condition overall survival of the patients (34).

The prolonged and sustained immune suppression needed for the management of cGVHD (between 1-3 years)(10), implies an increased risk of secondary infections and malignant processes, with an associated increase in mortality (4). The most prevalent secondary infections are of viral (HSV) and fungal origin (candidiasis), and exhibit unusual clinical manifestations and resistance to conventional therapy (38).

Hematological malignancies and lymphoproliferative disorders can be seen with some frequency and in early phases after HSCT. Secondary solid tumors are less common, though they increase over time after transplantation (11), with incidences of 2-6% after 10 years and 6-13% after 15 years (39). The mean time from transplantation to the development of malignancy is 7 years (1). One-third of all secondary malignancies affect the skin and oral cavity (39). There is a certain tendency to develop tumors of epithelial origin: one half of them are squamous cell carcinomas – this being the histological variant most often seen in the oral cavity (11,39). The characteristics of oral squamous cell carcinoma in patients with antecedents of HSCT are rather special: the lesions develop in younger individuals; with no male predilection; there is usually no history of smoking; the tongue and cheek mucosa are the most affected locations; and the lesions tend to recur and develop in multiple locations (39). Because of these complications and their late onset, long-term follow-up is particularly indicated in patients of this kind (11).

The use of biomarkers in cGVHD is the subject of different investigations and clinical trials. Unfortunately, no biomarker panel applicable to cGVHD has been developed to date. The similarities between the normal immune response and cGVHD possibly complicate the identification and application of such markers (18,35).

## Conclusions

Oral cGVHD is a frequent complication of HSCT. The diagnosis is usually established from the clinical findings, though a biopsy is sometimes required. The disorder manifests as lichenoid lesions, hyperkeratotic plaques and limited oral aperture secondary to sclerosis, and is usually mild. Locally applied corticosteroids are the treatment of choice and oral cGVHD is not regarded as a determinant factor for patient survival.
